# A rare case report of a Servelle-Martorell syndrome patient

**DOI:** 10.1016/j.ijscr.2023.108491

**Published:** 2023-07-08

**Authors:** Abraham Gita Ramanda Christanto, M. Ali Shodiq, Sahal Fatah, Wahyu Wiryawan

**Affiliations:** a Department of Surgery, Faculty of Medicine Universitas Diponegoro, Dr. Kariadi General Hospital, Semarang, Indonesia; b Division of Thoracic, Cardiac and Vascular Surgery, Department of Surgery, Faculty of Medicine Universitas Diponegoro, Dr. Kariadi General Hospital, Semarang, Indonesia

**Keywords:** Case report, Vascular malformations, Servelle-Martorell syndrome, Bone hypotrophy

## Abstract

**Introduction and importance:**

Servelle-Martorell syndrome (SMS) is a rare congenital anomaly that is frequently mistaken for Klippel-Trenaunay syndrome (KTS) or Parkes-Weber syndrome (PWS). SMS usually involves venous dilatations, soft tissue hypertrophy, and bone hypotrophy, while KTS and PWS usually have bone hypertrophy. The management of SMS is primarily conservative, and surgery should be done selectively. This study aimed to report a case of SMS and its management to relieve a painful aneurysm on the right knee by excision.

**Case presentation:**

A 16-year-old male patient presented with a slightly shorter right lower limb and multiple bluish swelling on his right lower limb. Supporting venography and angiography showed venous malformations, soft tissue hypertrophy, and bone hypotrophy on the right lower limb. The physical and supporting examinations led to the diagnosis of SMS. The patient was admitted because of severe pain in the right knee. Surgical excision of the venous malformation in the knee region was done to relieve the pain. The patient felt significantly reduced pain on one-month follow-up.

**Clinical discussion:**

SMS has similar features to KTS and PWS. The excision surgery was indicated due to severe pain in the right knee.

**Conclusion:**

SMS is a rare disease and important to be recognized as it is frequently mistaken as KTS or PWS. The management is primarily conservative and surgical management should only be done in severe aneurysmal complications and shunting. As venous malformations and pain can reoccur after surgical excision, regular follow-ups should be maintained.

## Highlights

• Servelle-Martorell syndrome (SMS) is a rare congenital anomaly and is frequently mistaken for Klippel-Trenaunay syndrome or Parkes-Weber syndrome.
• SMS usually involves venous dilatations, soft tissue hypertrophy, and bone hypotrophy.
• The management of SMS is primarily conservative and surgical management should only be done in severe aneurysmal complications and shunting.

## 1 Introduction

Servelle-Martorell syndrome (SMS) is a very rare congenital venous malformation [1]. This syndrome usually involves venous dilatation, soft tissue hypertrophy, and bone hypotrophy involving the upper or lower limbs [1]. SMS is frequently mistaken as other vascular malformations associated with other anomalies in the International Society for the Study of Vascular Anomalies (ISSVA) 2018 classification such as Klippel-Trenaunay syndrome (KTS) and Parkes-Weber syndrome (PWS). KTS and PWS also present similar symptoms to SMS but are characterized by bone hypertrophy [1,2]. When these syndromes are suspected, a complete history and physical examination should be performed carefully. Radiographs and contrast-enhanced imaging must be taken to help determine the diagnosis and assist in the management. Misdiagnosis or delayed diagnosis of SMS happens frequently due to the lack of knowledge of the syndrome.

These group anomalies are usually associated with specific gene mutations such as the PIK3CA gene in KTS and RASA1 gene in PWS [2]. The causal gene in the SMS has not been reported as it is very rare and not many studies have discussed it. Venous malformations in SMS may appear at birth and become more visible in childhood. A study by Weiss et al. mentioned that the shortening of the limb by bone hypotrophy may be caused by intraosseous vascular malformations which destruct the spongious and cortical bone [3]. No cure for the disease has been found for SMS, so the management regime is focused on symptomatic treatment to limit complications or disabilities and improve quality of life. The conservative management includes skincare and compression therapy [4]. Surgical management should restrictively be performed in aneurysmal complications or shunting and can be a definitive treatment on the indication [4]. In this study, we reported a rare case of a 16-year-old male patient with SMS features. Surgical management to relieve painful venous malformations on the right knee by excision was done at our hospital. This report was written in line with the SCARE criteria 2020 [5].

## 2 Case report

A 16-year-old Indonesian male patient was admitted to the emergency room of our hospital with a complaint of severe pain in his knee in the last three months that was not relieved with analgesics. The patient came from a low socio-economic family and was from a small district. At birth, the parents noticed a bluish lump on the right scrotum but did not consult any doctor. At 4 years old, a mass started to show on the right ankle of the patient. He underwent surgery to reduce the mass, but this did not result in a visible significant reduction. Since around that age, some parts of the right leg started to enlarge slowly and causing asymmetry. At 10 years old, the patient started to experience pain and underwent another surgery in the right thigh area and the pain subsided. After the last surgery, the patient had not experienced disturbing pain until three months before admission. Due to the lack of a prior definitive diagnosis, the patient was not advised to use compression garments daily and was only prescribed analgesics for pain.

**Fig. 1:**
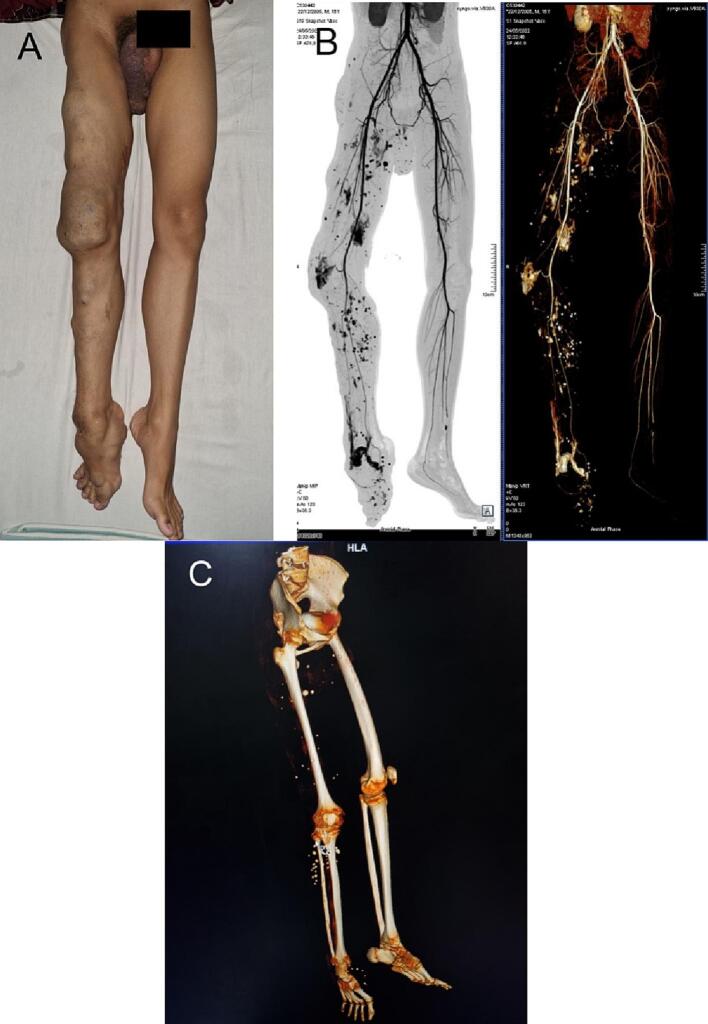
The preoperative condition of the patient. (A) The right lower limb was shorter than the left one. (B) The MSCT-angiography image showed venous aneurysms and phleboliths on the right lower limb. (C) The MSCT showed hypotrophy of the right lower limb with multiple phleboliths.

At the time of the admission, the physical examination showed multiple areas with bulging varicosities in a bluish color and soft tissue enlargement in the right lower limb, a slightly shorter right lower limb, and asymmetry (Fig. 1A). The right scrotum was also enlarged and bluish in color. The enlargement areas on the right lower limb were compressible, and the knee area was painful spontaneously and on palpation. The pain was aggravated when walking and lessened when lying down. The venography showed that the contrast filled the superficial-deep veins on the right foot, the superficial vein of the right leg, the right popliteal vein, and the right thigh superficial vein. These features were indicating aneurysmal enlargements. Vascular calcifications (phleboliths) of the soft tissue swellings were also found on the right thigh, knee, leg, and foot. The MSCT angiography showed multiple solid calcified lesions on the right scrotum, thigh, pelvis, and leg (Fig. 1B). Right bone hypotrophy (thigh, tibia, fibula) was also confirmed by MSCT (Fig. 1C). The physical and supporting examinations were like the features of KTS except that this patient had bone hypotrophy instead of bone hypertrophy. This led to the diagnosis of the rare SMS.

**Fig. 2:**
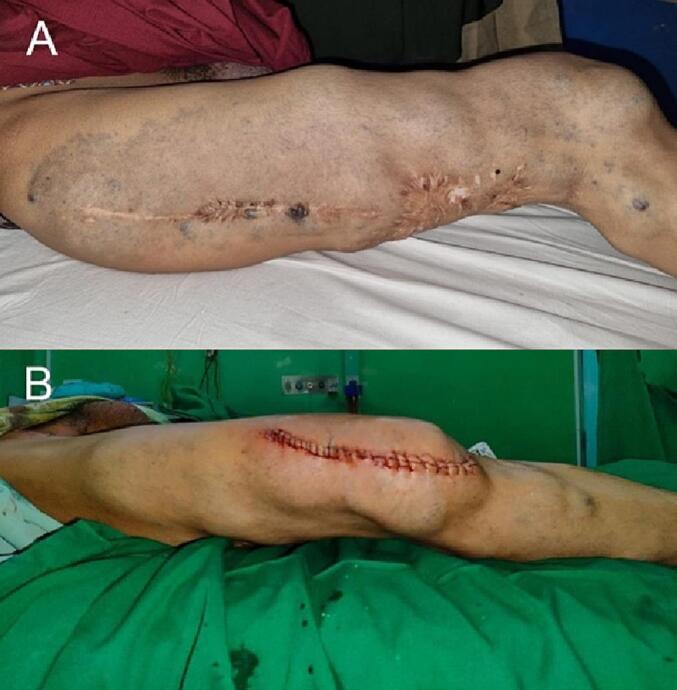
(A) The preoperative lateral view of the right thigh and knee region. (B) The postoperative image after the surgical excision of the venous malformations in the right knee region.

At our hospital, the patient was prepared for surgery. The patient underwent surgical excision of the venous malformations for pain and hypertrophy in the right knee region in a supine position (Fig. 2A, B) by a thoracic and cardiovascular surgeon. The patient was advised to wear elastic bandages postoperatively. During the one-month follow-up, the patient felt significantly reduced pain and was advised to use compression garments routinely to prevent trauma. Family consent for patient data and photographs was obtained. The patient was also scheduled for follow-ups. A multidisciplinary approach to this patient is required for better maintenance of the symptoms and to limit disabilities.

## 3 Discussion

Although both KTS and SMS are rare vascular disorders, KTS seemed to be more commonly known and the studies were more abundant than SMS. Two to five persons per 100,000 people are affected by KTS [6]. While also being infrequent, the prevalence and incidents of SMS have never been reported in studies before. This phenomenon may be influenced by the confusion and misdiagnosis of SMS into KTS due to similar symptoms and little information regarding SMS.

Port-wine stain from capillary malformations was a feature that occurs in 98 % of KTS patients [6]. The patient did not have a port-wine stain and only showed venous malformations without arteriovenous malformations. The MSCT of this patient also featured right limb hypotrophy. These signs eliminated the PWS and KTS as the differential diagnosis. Earlier, there were a few articles that proposed the concept of ‘inverse KTS’ that showed features of limb shortening instead of lengthening [7]. Despite the newly proposed inverse KTS, the signs and symptoms of our patient were more likely to lead to SMS which has been classified by ISSVA [2]. The aneurysmal enlargement caused by dilatation of superficial veins in SMS may create an atrocious appearance in the extremities as seen in the right lower extremity of our patient [8]. Multiple phleboliths were also reported to be featured in SMS patients as it was seen in this patient.

The therapy of SMS is primarily conservative, and surgery should not be done unless indicated. Surgery should be performed with the risks of bleeding, possible recurrence, and postoperative morbidity in mind. However, a study mentioned that avoiding surgery in some patients may bring risks of affecting deformities in other systems (i.e., digestive system, skeletal and muscular system, etc.) due to vascular malformations [8]. There are several studies associated with SMS and KTS that have reported successful surgical excision in reducing symptoms and limiting disabilities [9]. Severe pain was experienced by our patient which indicated the need for a more invasive treatment. This patient received excision surgery on the right knee to control the pain.

The patient had controlled bleeding and insignificant pain in his right knee after the surgery which confirmed the good result of the surgery. A study by Noel et al. reported 50 % of venous malformations recurrence after excision in KTS patients but clinical improvement was noted in most patients [10]. Due to the limited studies regarding SMS, the result of the previous study is valuable. With the high recurrence rate of venous malformations in mind, the patient should be recalled regularly to maintain the symptoms and monitor complications. Because SMS case is rare and complex, it requires a multi-disciplinary approach involving vascular surgeons, orthopedic surgeons, plastic surgeons, dermatologists, rehabilitation physicians, and physical therapists.

## 4 Conclusion

Servelle-Martorell syndrome is a rare disease and is important to be recognized as it is often mistaken as KTS or PWS. The management of SMS is primarily conservative, especially with compression garments. Surgical treatment is indicated for selected patients with severe pain or disability. The current patient had been having severe pain for three months and finally had it relieved after the surgery. The use of compression garments is important postoperatively and daily to reduce the risk of bleeding and trauma. Pain in SMS patients can reoccur due to recurrent varicose veins which may indicate another intervention in the future. Regular follow-ups should be done to prevent worsening symptoms of the patients. A multi-disciplinary approach should also be taken as this is a complex disorder.

## Consent for publication

Written informed consent was obtained from the patient's parents/legal guardian for publication and any accompanying images. A copy of the written consent is available for review by the Editor-in-Chief of this journal on request.

## Ethical approval

Ethical approval was waived by our institution.

## Funding

None.

## Guarantor

Abraham Gita Ramanda Christanto and M. Ali Shodiq.

## CRediT authorship contribution statement

Abraham Gita Ramanda Christanto: conceptualization, data collection, data analysis, manuscript writing-reviewing, and editing.
M. Ali Shodiq: conceptualization, performed surgery, data analysis, manuscript writing, and manuscript reviewing.
Sahal Fatah: manuscript writing and reviewing.
Wahyu Wiryawan: manuscript writing and reviewing.

## Declaration of competing interest

None.
